# Supplementary material for: Dynamics of the respiratory infectome in children with community-acquired pneumonia: insights from large and short time-scale analyses
Source: Microbiol Spectr. 2025 Oct 27;13(12):e01450-25. doi: 10.1128/spectrum.01450-25 (PMC12671164; doi:10.1128/spectrum.01450-25)
Supplement: Figures S1 and S2 — Fig. S1: Percentage of Reads in Each Library Percentage of reads in both metagenomic and meta-transcriptomic sequencing data of each library. Colors correspond to different types of reads. Fig. S2 Phylogenetic trees of detected viruses. Maximum likelihood phylogenetic trees are constructed using viral genomes or marker genes, such as capsid protein 1 (VP1), envelope glycoprotein gB (gB), and fiber protein (Fiber). All trees are midpoint-rooted for clarity. Red dots represent samples collected in 2022, while blue dots indicate samples from 2023. [file spectrum.01450-25-s0001.pdf]

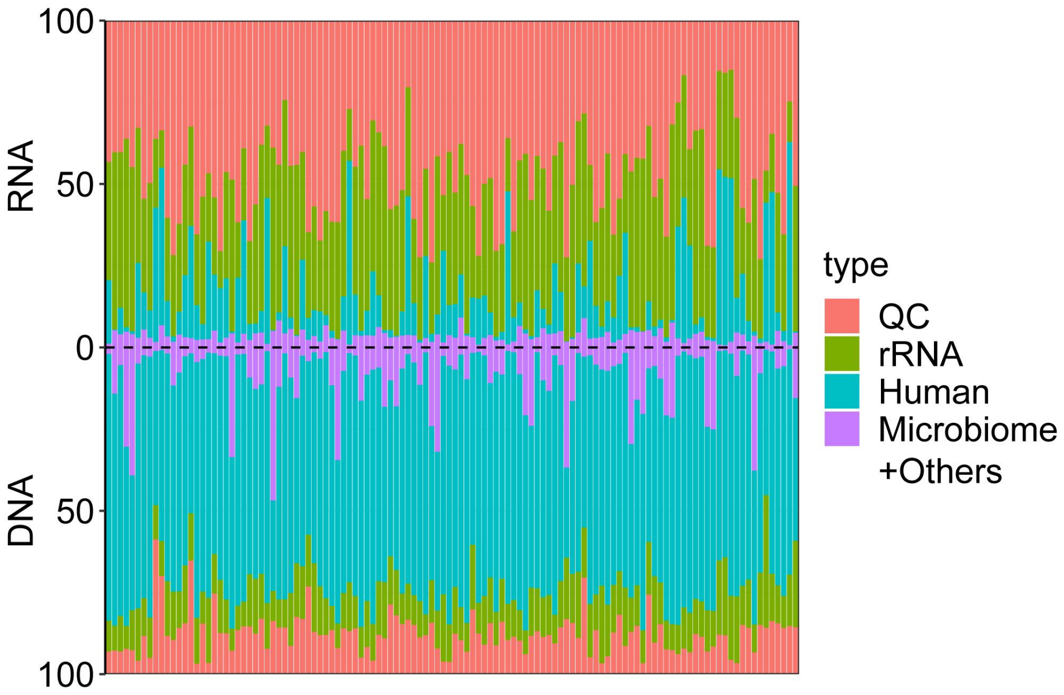

FIG S1 Percentage of Reads in Each Library Percentage of reads in both metagenomic and meta-transcriptomic sequencing data of each library. Colors correspond to different types of reads.

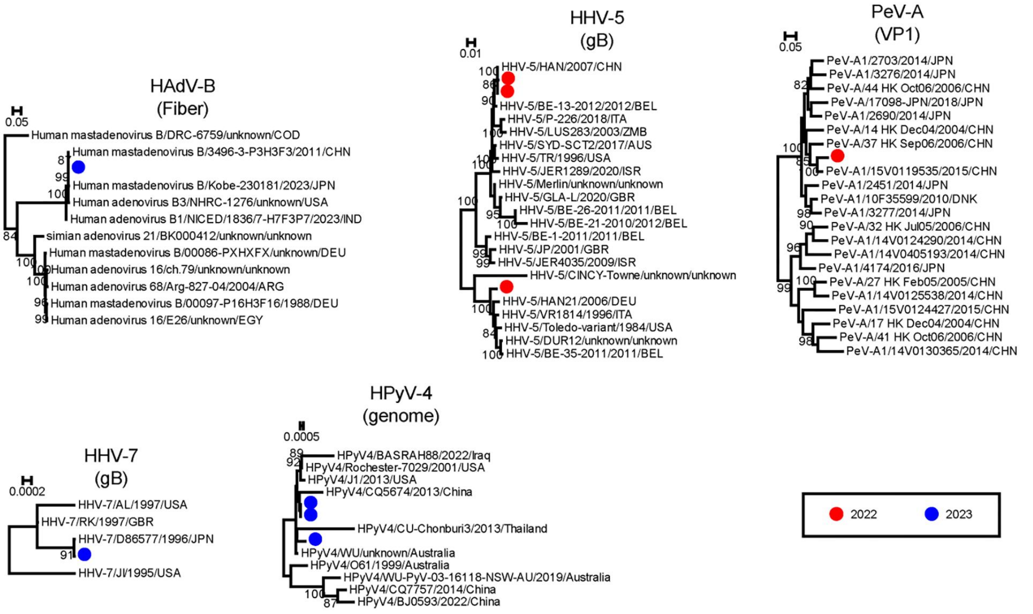

FIG S2 Phylogenetic trees of detected viruses. Maximum likelihood phylogenetic trees are constructed using viral genomes or marker genes, such as capsid protein 1 (VP1), envelope glycoprotein gB (gB), and fiber protein (Fiber). All trees are midpoint-rooted for clarity. Red dots represent samples collected in 2022, while blue dots indicate samples from 2023.
